# Retrospective observational study of diagnostic accuracy of the Xpert® MTB/RIF assay on fiberoptic bronchoscopy sampling for early diagnosis of smear-negative or sputum-scarce patients with suspected tuberculosis

**DOI:** 10.1186/1471-2466-14-137

**Published:** 2014-08-12

**Authors:** Pierre Le Palud, Vincent Cattoir, Brigitte Malbruny, Romain Magnier, Karine Campbell, Youssef Oulkhouir, Gérard Zalcman, Emmanuel Bergot

**Affiliations:** 1 CHU de Caen, Service de Pneumologie et Oncologie thoracique, Caen, F-14000, France; 2 CHU de Caen, Service de Microbiologie, Caen, F-14000, France; 3 Université de Caen-Basse Normandie, Caen, F-14000, France; 4 Groupe pour la Recherche et l’Enseignement en Pneumo-Infectiologie (GREPI) de la Société de Pneumologie de Langue Française (SPLF), Paris, F-75000, France

**Keywords:** Tuberculosis diagnosis, Fiberoptic bronchoscopy, Xpert® MTB/RIF assay, Smear microscopy

## Abstract

**Background:**

Fiberoptic bronchoscopy (FOB) is a useful diagnosis tool in low-burden countries for patients with suspected pulmonary tuberculosis (TB) who are smear-negative or sputum-scarce. This study sought to determine the accuracy of the Xpert® MTB/RIF (XP) assay using FOB samples.

**Methods:**

We retrospectively reviewed clinical, radiological, and microbiological characteristics of 175 TB-suspected patients requiring diagnostic FOB (bronchial aspirate or bronchoalveolar lavage) with XP assay. Polymerase chain reaction (PCR) and smear microscopy (SM) performances were first compared to culture, then to the final diagnosis, established based on clinical or radiological evolution when cultures were negative.

**Results:**

Of the total 162 included patients, 30 (18.5%) had a final diagnosis of pulmonary TB, with positive cultures reported in 23. As compared to culture, sensitivity and specificity values were 80.0% and 98.6% for the XP assay, and 25.0% and 95.8% for SM, respectively. As compared to final diagnosis, the corresponding performance values were 60.0% and 100.0% for the XP assay, and 16.7% and 95.5% for SM, respectively. The sensitivity of the XP assay was significantly higher than that of SM in both cases (*p* = 0.003 and *p* = 0.001). Concerning the final diagnosis, both XP assay and culture sensitivities were similar (60% *vs.* 66.7%). PCR assay enabled pulmonary TB to be diagnosed earlier in 13 more cases, compared to SM.

**Conclusion:**

Our study has confirmed the clinical benefits provided by XP assay compared to SM for the early diagnosis of suspected pulmonary TB cases requiring FOB, on *per* procedure samples, especially in a low TB-burden country.

## Background

Tuberculosis (TB) still constitutes a major health problem worldwide, with an 8.8% incidence and 1.3% mortality rate reported in 2010 [1]. In the same year, TB incidence was reported at 8.1 cases per 100,000 in France [2]*,* defining it as a low TB-burden country. It is worth noting that 73% of TB cases were pulmonary infections, with almost 50% producing a negative smear microscopy (SM) [2].

By detecting active pulmonary TB early, an appropriate treatment can be initiated, and disease transmission can then be preemptively blocked. Some patients presenting with active pulmonary TB may, however, exhibit negative sputum acid-fast bacilli (AFB) smears. In developed countries, fiberoptic bronchoscopy (FOB) is considered a good option for these cases that pose a diagnostic challenge [3], although SM is still exhibiting low sensitivity on FOB samples, with 5-35% on bronchial aspirates (BA) and 10-30% on bronchoalveolar lavages (BAL) [4-9]. Furthermore, while mycobacterial culture remains the gold standard for laboratory diagnosis of TB, it requires 2–6 weeks to confirm a diagnosis. This results in delays in initiating appropriate treatment while waiting for this confirmation, except for cases where there is strong enough clinical suspicion to initiate a presumptive anti-TB therapy.

Several polymerase chain reaction- (PCR-) based molecular methods have recently been developed for early TB diagnosis and rapid detection of drug resistance from clinical specimens [10-14]. The Xpert® MTB/RIF assay (Cepheid, Sunnyvale, CA, USA) is one of these methods, and consists of a hemi-nested real-time PCR test that simultaneously identifies *Mycobacterium tuberculosis* and detects rifampicin resistance, as a surrogate of multidrug resistance (MDR), directly from clinical specimens. This assay requires less than 2 hours, and its key advantage over other PCR methods is that it is a fully-automated process, designed to run on the GeneXpert Dx system (Cepheid). This system incorporates DNA extraction, often considered the critical step [15], along with real-time PCR amplification and detection in a single hands-free process, thus acting as a real “lab-on-chip” device.

Since December 2010, WHO has recommended the Xpert® MTB/RIF assay as a *bona fide* follow-on test due to its high-quality performance [16], compared to microscopy, whenever MDR-TB or HIV are of lesser concern, and especially in cases of smear-negative specimens [17]. This conditional recommendation does, in fact, exclusively concern sputum samples [17], whereas no specific recommendations for FOB samples have yet been formulated. Finally, there have only been two recent studies that have assessed the Xpert® MTB/RIF assay performance using FOB samples for TB diagnosis in high TB-burden countries [18,19].

This study sought to evaluate the clinical value of the Xpert® MTB/RIF assay using FOB samples for an early diagnosis of pulmonary TB in either patients with negative sputum AFB smears or those who could not produce an expectorated sputum sample. Patients were treated in a French university hospital in a low TB-burden region (5.9 cases per 100,000 in 2010).

## Methods

### Study population

We retrospectively reviewed the medical records of patients with suspected TB requiring a diagnostic FOB at the Caen University Hospital (Basse-Normandie region, North-Western France) from October 2009 to April 2013. TB was suspected based on clinical features (*e.g.,* cough, hemoptysis, fever, asthenia, loss of weight, and night sweats) or radiological features (*e.g.,* nodule, pneumonia, excavation, and pleurisy). All included patients either produced a negative sputum AFB SM prior to FOB procedure or were unable to produce sputum.

Our institution, a regional center of reference for TB diagnosis and treatment, has adopted the Xpert® MTB/RIF assay since 2009. Patients were included in the ongoing retrospective survey if one or more Xpert® MTB/RIF assays had been performed on their FOB samples, in addition to SM and mycobacterial culture procedures. Samples were excluded if culture results were unavailable or if the Xpert® MTB/RIF assay produced invalid results (see below). Patients who had previously received anti-TB drugs were also excluded from analysis.

### Bronchoscopic procedures

Bronchoscopy was performed using a flexible fiberscope of either 4.9 mm (model BF-P180, Olympus Optical) or 5.1 mm in diameter (model F1-16RB, Pentax) by trained lung specialists who first inspected the bronchial tree and then collected BA or BAL specimens. The type of sample was chosen at the specialist’s discretion and depending on the patient’s tolerance of the procedure. The lung section sample was generally chosen based on chest X-ray or CT-scan abnormalities. BA samples were obtained by the aspiration of pure bronchial secretions or following the instillation of 20-50 mL isotonic saline solution. For the BAL samples, 100-150 mL isotonic saline solution was instilled by 50 mL aliquots in a lung segment, and then aspirated.

### Microbiological diagnosis

All samples were digested, decontaminated by means of N-acetyl-cysteine-2% NaOH, concentrated by centrifugation (at 3,500 rpm for 20 min), and tested with SM, culture (used as the reference technique), and PCR.

For the smear test, fixed preparations were stained with auramine and visualized under a fluorescence microscope (at × 400 magnification). Each slide was observed for 5 min, corresponding to 200 fields examined. Sample aliquots of 500 μL and 200 μL were inoculated in an MGIT liquid medium (BD Diagnostics, Le Pont-de-Claix, France) or on Coletsos slants (Bio-Rad, Marnes-la-Coquette, France), respectively. Liquid cultures were automatically monitored by the BACTEC MGIT 960 system (BD Diagnostics) for up to 6 weeks, while solid media were studied for up to 12 weeks. Positive cultures were those with the presence of *M. tuberculosis* confirmed by means of the TB Ag MPT64 Rapid® assay (Standard Diagnostics, Yongin, South Korea), and rifampicin susceptibility was tested using the MGIT 960 SIRE kit (BD Diagnostics) [20]. The different species of nontuberculous mycobacteria (NTM) were identified using the GenoType® *Mycobacterium* CM/AS (HainLifescience, Nehren, Germany).

For the Xpert® MTB/RIF assay, a 500 μL aliquot was poured into a single-use disposable cartridge that was placed into the GeneXpert™ Dx module, with the results produced in less than 2 hours. Each PCR run comprised an internal control for sample processing (DNA extraction) and PCR validity (presence of inhibitors), with positive and negative controls tested every day. The system automatically interpreted all results from measured fluorescent signals, with embedded calculation algorithms, into the following categories: invalid, if PCR inhibitors were detected with amplification failure; negative or positive. If positive, the strain was categorized as susceptible or resistant to rifampicin [21,22].

### Pulmonary TB diagnosis

The final diagnosis of active pulmonary TB was primarily based on the *M. tuberculosis* culture taken from a respiratory specimen. Additional cases were classified as definitive TB, taking into account both the clinical symptoms and histological/radiological findings compatible with active pulmonary TB, as well as improvement observed with anti-TB specific therapy.

### Ethical considerations

According to the WHO recommendations on the Xpert® MTB/RIF assay [16], the use of this diagnosis TB test has been routinely implemented in our University hospital. According to French laws, a formal agreement from an ethics committee is not required for retrospective collection of data dealing with usual standard medical care *(ref: law n°2004-806 from August, 9th 2004, modified by government ordinance n°2066-477 from April 26th 2006, article R1121-3, Journal officiel de la République Française)*. All collected data from the charts of the Microbiology Department were anonymous and therefore complied with the restrictive requirements of the *Commision Nationale de l’Informatique et des Libertés (CNIL)*, the organization that ensures the application of data privacy laws in France. Moreover, the study protocol was evaluated and approved by the institutional review board of the French Society for Respiratory Medecine (“Société de Pneumologie de Langue Française”) for observational studies (CEPRO). Finally, all patients were informed of our TB diagnostic strategy, received written information about the FOB procedure and gave there oral consent (as recommended by the French Society of Respiratory Diseases).

### Statistical analysis

Sensitivity, specificity, positive predictive value (PPV), and negative predictive value (NPV) were calculated, along with the corresponding 95% confidence intervals. Sensitivity and specificity values were compared by means of the McNemar’s test. A *p*-value inferior to 0.05 was considered statistically significant. Statistical analysis was performed using the SPSS Statistics 20.0 software (Armonk, NY, USA).

## Results

### Patient characteristics

From October 2009 to April 2013, 175 consecutive patients presenting with suspected active pulmonary TB underwent FOB with an Xpert® MTB/RIF assay using respiratory samples. A total of 13 samples from 13 different patients were excluded from our study for the following reasons: 1) two due to PCR failure; 2) 10 due to unavailable culture; 3) one due to recent anti-TB treatment. A total of 229 FOB samples taken from 162 patients (median age: 54 years; male/female ratio: 1.7) were included.

The principal clinical symptoms were cough (51.9%) and general symptoms, such as asthenia, loss of appetite, and loss of weight for half of the patients (Table 1). The most common lesions detected by chest imaging were nodules (CT-scan: 60.5%) and pneumonia (CT-scan: 30.2%) (Table 1). It should be noted that 18, 9, and 80 patients had 1, 2, and 3 sputum samples, respectively, that were collected before FOB, with the number of Xpert® MTB/RIF assays performed on the sample ranging from 0 to 3 for each patient (0: 110 patients; 1: 21; 2: 22; 3: 9). No sputum could be collected before the FOB procedure for 55 patients (34%) due to their inability to expectorate sputum.

**Table 1:** Demographic, clinical, and radiological characteristics of the 162 included patients

**Characteristic**	**N (%)**
**Median age, years (range)**	**54 (34–74)**
Gender	
Male	102 (63.0)
Female	60 (37.0)
HIV	
Positive	7 (4.3)
Negative	98 (60.5)
NA	57 (35.2)
Symptoms	
Cough	84 (51.9)
Hemoptysis	17 (10.5)
General symptoms^a^	73 (45.1)
Fever	43 (26.5)
Night sweats	26 (16.0)
NA	11 (6.8)
Chest radiography	
Nodules	87 (53.7)
Pneumonia	44 (27.1)
Excavation	14 (8.6)
Pleurisy	21 (13.0)
NA	13 (8.0)
Chest CT scan	
Nodules	98 (60.5)
Pneumonia	49 (30.2)
Excavation	27 (16.7)
Pleurisy	21 (13.0)
NA	29 (17.9)
Final diagnosis	
Pulmonary Tuberculosis	30 (18.5)
Pleural Tuberculosis	2 (1.2)
Tuberculosis sequelae	11 (6.8)
NTM^b^ infection	9 (5.6)
Non-mycobacterial lung infection^c^	28 (17.3)
Bronchial disease exacerbation	7 (4.3)
Non-mycobacterial granulomatosis disease	7 (4.3)
Lung cancer	21 (13.0)
Other^d^	23 (14.2)
No diagnosis	18 (11.1)
NA	6 (3.7)

^a^General symptoms: asthenia and/or loss of appetite and/or loss of weight.

^b^*NTM*: Nontuberculous mycobacteria (including four *M. avium*, two *M. xenopi*, one *M. szulgai*, one *M. malmoense*, and one other).

^c^Including 24 infectious pneumonia, two lung abscesses, and two invasive aspergillosis.

^d^Including eight non-infectious pneumonia, five acute pulmonary edema, five idiopathic hemoptysis, two interstitial lung disease exacerbations, one pulmonary infarction, one intra-alveolar hemorrhage, and one inflammatory pseudo-tumor lung.

**Figure 1:**
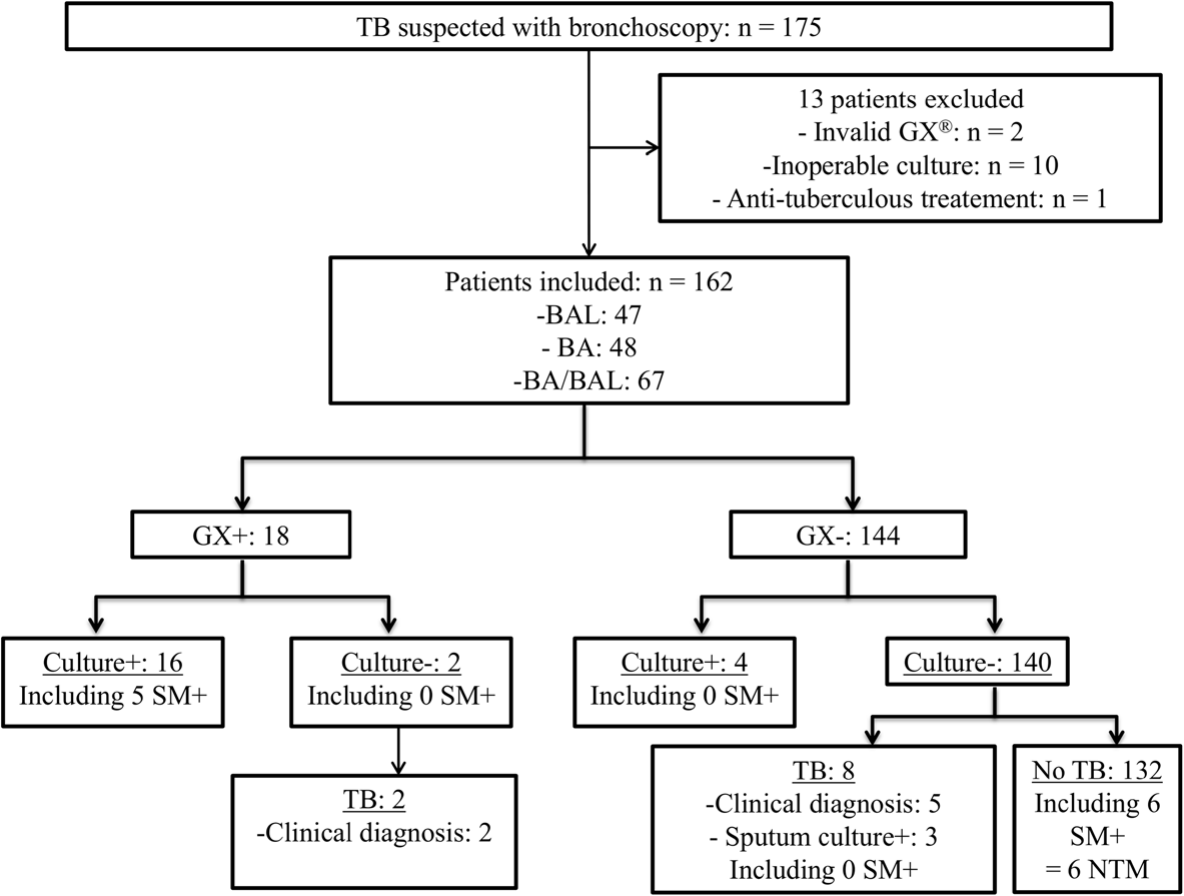
**Flow diagram of patients included in the study.** BA: bronchial aspirate; BAL: bronchoalveolar lavage; SM: smear microscopy; GX: Xpert®MTB/RIF assay; NTM: nontuberculous mycobacteria; TB: tuberculosis.

Xpert® MTB/RIF assay was performed on BA (n = 48), BAL (n = 47), or a BA/BAL mix (n = 67) (Figure 1). Out of the 162 patients, 30 TB cases were finally diagnosed, corresponding to a prevalence of 18.5% (Figure 1). Of these cases, positive cultures were reported on FOB and sputum samples for 20 and 3, respectively, including one TB case caused by *“Mycobacterium canetti”*. For the remaining seven patients, diagnosis was based on clinical and histological/radiological features, with favorable evolution under anti-TB therapy serving as confirmation. The final diagnoses for the other patients are detailed in Table 1.

### Performances of microbiological methods

In comparison with culture used as reference, the overall sensitivity, specificity, PPV, and NPVs for the Xpert® MTB/RIF assay were 80.0% (95% CI: 57.8-92.5), 98.6% (95% CI: 94.7-99.9), 88.9% (95% CI: 66.0-98.1), and 97.2% (95% CI: 92.8-99.2), respectively. The corresponding values for SM were recorded as 25.0% (95% CI: 10.8-47.3), 95.8% (95% CI: 90.9-98.3), 45.5% (95% CI: 21.3-72.0), and 90.1% (95% CI: 84.2-94.0), respectively (Table 2). It is interesting to note that the sensitivity of the Xpert® MTB/RIF assay was found to be significantly higher than that of SM (*p* = 0.003; Table 2).

**Table 2:** Performances of the Xpert®MTB/RIF assay, smear microscopy, and culture using FOB samples for the diagnosis of pulmonary tuberculosis

	**Performances relative to culture**		**Performances relative to final diagnosis**	
	**Sensitivity%**	**Specificity%**	**Sensitivity%**	**Specificity%**
	**(95% CI) n**	**(95% CI) n**	**(95% CI) n**	**(95% CI) n**
Xpert® MTB/RIF assay	80.0	98.6	60.0	100.0
	(57.8-92.5)	(94.7-99.9)	(42.3-75.4)	(96.6-100.0)
	16/20	140/142	18/30	132/132
Smear microscopy	25.0	95.8	16.7	95.5
	(10.8-47.3)	(90.9-98.3)	(6.9-34.0)	(90.2-98.1)
	5/20	136/142	5/30	126/132
Culture	-	-	66.7	100.0
			(48.7-80.9)	(96.6-100.0)
			20/30	132/132
*p*-value^a^	*0.003*	*0.288*	*0.001*^*b*^	*0.041*^*b*^
			*0.683*^*c*^	*-*
			*0.0003*^*d*^	*0.041*^*d*^

^a^*p*-values were calculated using the McNemar’s test.

^b^*p*-value between Xpert®MTB/RIFassay *vs.* smear microscopy.

^*c*^*p*-value between Xpert®MTB/RIFassay *vs.* culture.

^*d*^*p*-value between culture *vs.* smear microscopy.

When considered relative to the final diagnosis, the performances of both sensitivity (60%; 95% CI: 42.3-75.4) and specificity (100%; 95% CI: 96.6-100.0) of the Xpert® MTB/RIF assay were also shown to be significantly higher than those of SM (16.7%; 95% CI: 6.9 to 34.0; 95.5%; 95% CI: 90.2 to 98.1) (*p* = 0.001 and *p* = 0.041) (Table 2). PPV and NPV were 100.0% (95% CI: 79.3-100.0) and 91.7% (95% CI: 85.9-95.3) for the Xpert® MTB/RIF assay, respectively, while these values were measured at 45.5% (95% CI: 21.3-72.0) and 83.4% (95% CI: 76.6-88.6) for SM, respectively. It is worth noting that all six samples with a positive SM and a negative PCR assay were seen to grow an NTM species. Regarding the final diagnosis, no significant difference was observed between Xpert® MTB/RIF assay and culture sensitivities (60%; 95% CI: 42.3-75.4 *vs.* 66.7%; 95% CI: 48.7-80.9; *p* = 0.683).

The Xpert® MTB/RIF assay also enabled earlier TB diagnosis and treatment for 13 more patients than was possible with SM (Table 3). In addition, the Xpert® MTB/RIF assay led to the early detection of one MDR-TB case in our series, which was subsequently confirmed using phenotypic tests.

**Table 3:** Gain in early pulmonary tuberculosis diagnosis with the Xpert® MTB/RIF assay

**N = 162 patients**	**TB + (N = 30 patients)**	**TB- (N = 132 patients)**
Xpert®MTB/RIF (XP)+	18	0
Smear microscopy (SM) +	5	6 (=6 NTM^a^)
Gain in early TB^b^ diagnosis (XP-SM)	13	0

^a^*NTM*: Nontuberculous mycobacteria.

^b^*TB*: Tuberculosis.

## Discussion

FOB constitutes an interesting alternative for TB diagnosis in smear-negative or sputum-scarce patients, especially in developed countries where this procedure is widely available. Given that WHO recommendations on the Xpert® MTB/RIF assay only pertain to sputum samples [17], further investigation must be conducted regarding the use of this PCR on FOB samples. To the best of our knowledge, there has been no publication specifically describing the clinical interest and accuracy of the Xpert® MTB/RIF assay using FOB samples for TB diagnosis in a low-prevalence country.

Our study produced results in line with previous reports on respiratory samples [16,23-31], demonstrating that the Xpert® MTB/RIF assay significantly outperformed SM on FOB samples (sensitivities: 80.0% *vs.* 25.0%; *p* = 0.003) in a low TB-prevalence area. The sensitivity of the PCR assay was found to be lower when it was calculated relative to the final diagnosis, yet still remained significantly higher than that achieved with SM (60.0% *vs.* 16.7%; *p* = 0.001). Our findings revealed similar performances to those reported by two recently published studies, which had previously explored the clinical usefulness of the Xpert® MTB/RIF assay on FOB samples, although this was conducted in high TB-prevalence areas [18,19]. In the Lee *et al.* retrospective study, 132 patients were recruited in a single South-Korean center. The study reported sensitivity and specificity values (relative to the culture) of 81.6% and 100.0% for the Xpert® MTB/RIF assay, compared to 13.2% and 98.8% for SM, respectively [18]. Theron *et al.* prospectively included 154 patients in a South-African single-center study and specifically analyzed BAL samples. The resulting sensitivity and specificity values compared to the culture were 92.6% and 96.0% for the Xpert® MTB/RIF assay, and 57.7% and 99.3% for SM, respectively [19].

We can therefore confirm that for suspected TB requiring bronchoscopic procedure, the Xpert® MTB/RIF assay is a potential alternative to SM using FOB samples. In our study, PCR allowed for early pulmonary TB diagnosis to be established, enabling appropriate treatment to be started early for 13 more patients as compared to SM (72%) (*p* < 0.001; Table 3). In these patients, the definitive TB diagnosis was secondarily confirmed either by culture or clinical evolution under specific antimicrobial therapy. These findings were similar to those of the Theron *et al.* study (>80%) [19], and are of particular significance in a low TB-incidence area, where there is a higher proportion of alternative diagnosis, such as lung cancer or pyogenic bacterial pneumonia.

Our study objectives did not include assessing the value of the GeneXpert® assay in detecting rifampicin resistance on FOB samples, since the incidence of such resistance was expected to be low, as seen in previous studies [18,19]. The unique case of MDR-TB was, however, unambiguously detected, and all PCR results were corroborated by those that were phenotypically determined.

Our study presented some limitations. Firstly, this was a retrospective study, despite all Xpert® MTB/RIF assays being performed on fresh FOB samples. Secondly, it was a single-center study. Finally the PCR assays were not systematically performed on the same FOB sample for all patients (BA or BAL). A prospective study should be designed with systematized and parallel FOB sampling (BA, BAL). This would provide a more proficient way of testing the Xpert® MTB/RIF assay in different types of per-endoscopic respiratory samples, an endpoint that we were unable to analyze due to our retrospective design. Cost-effectiveness studies should also be conducted in order to specify the rational use of the Xpert® MTB/RIF assay on FOB samples in novel diagnostic algorithms, including this PCR assay in well-resourced countries.

## Conclusion

In summary, our study confirms the clinical usefulness of the Xpert® MTB/RIF assay, compared to SM, for the early diagnosis of suspected pulmonary TB requiring FOB, performed on *per* procedure samples. This is a reproducible commercial assay, and should especially be considered as a relevant option in low TB-burden countries.

## Competing interests

The authors declare that they have no competing interests.

## Authors’ contributions

Study conception and design: PLP, VC, GZ, and EB. Acquisition of data: PLP, RM, KC and YO. Drafting of manuscript: PLP, VC, GZ, and EB. Analysis and interpretation of data: BM. Critical revision: VC, GZ, and EB. All authors read and approved the final manuscript.

## Pre-publication history

The pre-publication history for this paper can be accessed here:

http://www.biomedcentral.com/1471-2466/14/137/prepub
